# INSIGHTS FOR IMPROVING OLDER ADULT NUTRITION WITH MULTIVITAMIN/MINERAL SUPPLEMENTS

**DOI:** 10.1093/geroni/igae098.4008

**Published:** 2024-12-31

**Authors:** Roger Fielding, Robert Blancato, Kathryn Porter Starr, Shelley McDonald, Rachel Zimmer, Jennifer Pettis, Karen Tracy

**Affiliations:** Jean Mayer USDA Human Nutrition Research Center on Aging at Tufts University, Boston, Massachusetts, United States; National Association of Nutrition and Aging Services Programs (NANASP), National Association of Nutrition and Aging Services Programs (NANASP), District of Columbia, United States; Duke University School of Medicine, Hillsborough, North Carolina, United States; DUKE UNIVERSITY MEDICAL CENTER, Durham, North Carolina, United States; Wake Forest Baptist Health, Wake Forest Baptist Health, North Carolina, United States; Gerontological Society of America, Clifton Park, New York, United States; Gerontological Society of America, Gerontological Society of America, District of Columbia, United States

## Abstract

In June 2024, GSA convened an expert roundtable to address the role of multivitamin/mineral supplements (MVMs) as part of complete nutrition for older adults. Older adults often do not obtain adequate amounts of micronutrients from their regular dietary patterns and are at risk for nutritional deficiencies. Reasons cited by experts included physiologic changes; comorbid conditions; conditions associated with aging (e.g., sarcopenia, frailty); socioeconomic factors; declines in physical function; and interrelated changes in cognition, functional capacity, mental health, and mobility. Nutrition has wide-ranging effects on health and well-being, and nutrition screening would aid in identifying deficiencies. However, nutritional status is not consistently assessed and documented in clinical practice. Experts proposed expanding interdisciplinary care and expanded payment opportunities as strategies to improve nutritional assessments and identified emerging care models that successfully address the nutritional needs of older adults. Expanded education and training opportunities related to nutrition for clinicians and implementing strategies for effectively educating older adults about nutrition were also cited as important needs. Experts noted that MVMs may be recommended to help meet nutritional needs in older adults. Providers should consider polypharmacy, potential medication/MVM interactions, and chronic conditions when making recommendations, and educational supports are needed to enhance acceptance of and adherence to recommendations. Experts also identified strategies for enhancing nutritional research and incorporating nutritional information into clinical practice guidelines to increase the clinician confidence around MVM recommendations.

